# CPPU may induce gibberellin-independent parthenocarpy associated with *PbRR9* in ‘Dangshansu’ pear

**DOI:** 10.1038/s41438-020-0285-5

**Published:** 2020-05-01

**Authors:** Liu Cong, Ting Wu, Hanting Liu, Huibin Wang, Haiqi Zhang, Guangping Zhao, Yao Wen, Qianrong Shi, Lingfei Xu, Zhigang Wang

**Affiliations:** College of Horticulture, Northwest A&F University, Taicheng, Road No.3, Yangling, Shaanxi, Province China

**Keywords:** Cytokinin, Fruiting

## Abstract

Parthenocarpy is a valuable trait in self-incompatible plants, such as pear. *N*-(2-chloro-4-pyridyl)-*N’*-phenylurea (CPPU), a synthetic cytokinin analog, can induce parthenocarpy in pear (*Pyrus* spp.), but the mechanism of induction is unclear. To investigate the role of gibberellin in CPPU-induced parthenocarpy in pear, CPPU supplemented with paclobutrazol (PAC) was sprayed onto ‘Dangshansu’ pear. We found that the fruit set rate of pear treated with CPPU supplemented with PAC was identical to that in a CPPU-alone treatment group. In regard to cell development, CPPU mainly promoted hypanthium cell division and expansion, and PAC application had no influence on CPPU-induced cell development. RNA sequencing revealed that *gibberellin 20 oxidase* and *gibberellin 3 oxidase* genes were not differentially expressed following CPPU treatment. According to the analysis of fruit phytohormone content, the CPPU treatments did not induce gibberellin biosynthesis. These results suggest that CPPU-induced parthenocarpy may be gibberellin independent in ‘Dangshansu’ pear. After CPPU treatment, the indole acetic acid (IAA) content in fruit was significantly increased, and the abscisic acid (ABA) content was significantly decreased. Similarly, RNA sequencing revealed that many genes involved in the auxin and ABA pathways were significantly differentially expressed in the CPPU treatment groups; among them, *indole-3-pyruvate monooxygenase* (*YUCCA*) was significantly upregulated and *9-cis-epoxycarotenoid dioxygenase* (*NCED*) was significantly downregulated. IAA and ABA may thus play important roles in CPPU-induced parthenocarpy. *PbTwo-component response regulator9* (*PbRR9*), *PbYUCCA4*, and *PbNCED6* were then selected to further elucidate the mechanism of CPPU-induced parthenocarpy. A yeast one-hybrid assay indicated that *PbRR9* can combine with the *PbYUCCA4* and *PbNCED6* promoters. Dual luciferase assays revealed that *PbRR9* can promote and repress the activities of the *PbYUCCA4* and *PbNCED6* promoters, respectively. After the transient expression of *PbRR9* in fruits, *PbYUCCA4* expression was significantly upregulated, and *PbNCED6* expression was significantly downregulated. This study uncovered a CPPU-induced parthenocarpy mechanism that is different from that in tomato. CPPU may upregulate *PbYUCCA4* and downregulate *PbNCED6* by upregulating *PbRR9*, thereby increasing IAA content and decreasing ABA content to ultimately induce parthenocarpy in ‘Dangshansu’ pear. However, because only a single time point was used and because ‘botanical’ and ‘accessory’ fruits have different structures, this conclusion is still preliminary.

## Introduction

Fruit set, which generally requires successful pollination and fertilization, initiates fruit development^1,2^. Pollination and subsequent fertilization are sensitive to environmental conditions^3^, such as low temperatures. Parthenocarpy is the phenomenon in which an unfertilized ovary develops into a seedless fruit^4^. Pear is naturally self-incompatible and requires commercial producers to plant pollinizer trees to achieve adequate fertilization and fruit set. Understanding the mechanisms of parthenocarpy is beneficial, as it offers the potential to reduce production costs and increase fruit yield.

Parthenocarpy is closely related to the coordinated action of different hormones produced in unpollinated ovaries^5^. Following successful pollination and fertilization, auxin synthesis is activated in the ovary, and the endogenous auxin content significantly increases to a level sufficient to induce fruit development^6^. Auxin was the first phytohormone discovered to induce parthenocarpy in plants^7,8^. Previous studies have indicated that an ectopic supply of auxin or auxin-like substances can induce parthenocarpy in strawberry^9^, tomato^10^, pear^11,12^, and eggplant^13^. In addition, changes in auxin biosynthesis and signaling pathways have been shown to induce parthenocarpic fruit set and fruit development. In tobacco and eggplant, the overexpression of the ovule-specific auxin biosynthesis gene *DefH9::tryptophan 2-monooxygenase (iaaM)* results in parthenocarpy^14^. When overexpressed in tomato, the auxin receptor *transport inhibitor response* (*TIR*) is responsible for parthenocarpic fruit^15^. The downregulation of the negative regulator *auxin response factor7 (ARF7)* in tomato leads to parthenocarpic fruit development, thus indicating that the reduced expression of indole acetic acid (IAA) repressor genes can result in parthenocarpy^16^. Similarly, *indoleacetic acid-induced protein 9* (*SlIAA9*)-downregulated transgenic tomato lines also present a parthenocarpic phenotype^17,18^. Gibberellin is another important hormone that contributes to parthenocarpy. The exogenous application of gibberellins can induce fertilization-independent fruit development in apple^19^, rose^20^, loquat^21^, grape^22^, and pear^23,24^. The gibberellin content in plants is regulated by the expression of gibberellin oxidase (GAox)^25^, whose influence has also been implicated in the occurrence of parthenocarpy. In tomato, overexpression of *gibberellin 20-oxidase* and silencing of *gibberellin 2-oxidase* can both lead to the production of parthenocarpic fruits in the absence of pollination^26,27^. Similar to auxin-related parthenocarpy, RNAi silencing of DELLA—the key repressor of the gibberellin signaling pathway—can induce parthenocarpic fruit development^28^. Crosstalk between auxin and gibberellin has been reported in tomato and pear, with auxin being upstream of gibberellin in parthenocarpy^10,12^.

In addition to auxin and gibberellin, cytokinin induces parthenocarpy in horticultural plants^29–32^, but the mechanism of cytokinin-induced parthenocarpy is not understood. Although *N*-(2-chloro-4-pyridyl)-*N’*-phenylurea (CPPU), a synthetic cytokinin analog, induces parthenocarpic fruit development in pear^23^, the mechanism of CPPU-induced parthenocarpy in this species has not been clearly elucidated. In this study, we sprayed ‘Dangshansu’ pear with CPPU to induce parthenocarpy and then investigated the underlying mechanism.

## Results

### CPPU may induce GA-independent parthenocarpy in ‘Dangshansu’ pear

In the hand-pollinated group, the fruit set rate reached 83.8% (Table 1). The fruit set rate of the CPPU treatment group was 93.5%, which was significantly higher than that of the pollinated group (Table 1). Because fruit set is an important and fundamental process in parthenocarpy, these results suggest that CPPU may induce parthenocarpy in ‘Dangshansu’ pear. To investigate the role of gibberellin (GA) in CPPU-induced parthenocarpy, we treated flowers with CPPU supplemented with PAC, a GA biosynthesis inhibitor. We found that PAC-supplemented CPPU still led to parthenocarpy in ‘Dangshansu’ pear; the fruit set rate reached 94.3%, which was not significantly different from that in the CPPU-alone treatment group (Table 1). We thus speculated that CPPU induces parthenocarpy in ‘Dangshansu’ pear that may be independent of GA.

**Table 1:** The effects of *N*-(2-chloro-4-pyridyl)-*N’*-phenylurea (CPPU) and paclobutrazol (PAC) on ‘Dangshansu’ pear fruit set

Treatment	UP	P	CPPU	CPPU + PAC
Fruit set rate (%)	0c	83.8 ± 2.58b	93.5 ± 1.56a	94.3 ± 1.28a

*UP* unpollinated, *P* pollinated, *CPPU* treatment with CPPU alone, *CPPU* *+* *PAC* treatment with PAC-supplemented CPPU. Significant differences among treatments as determined by one-way ANOVA (*P* < 0.05) are indicated using different lowercase letters (a, b, c). Error bars represent the standard deviation of the mean (SD; *n* = 3)

### Morphological and histological observations of CPPU and CPPU + PAC-induced parthenocarpic fruits

**Fig. 1:**
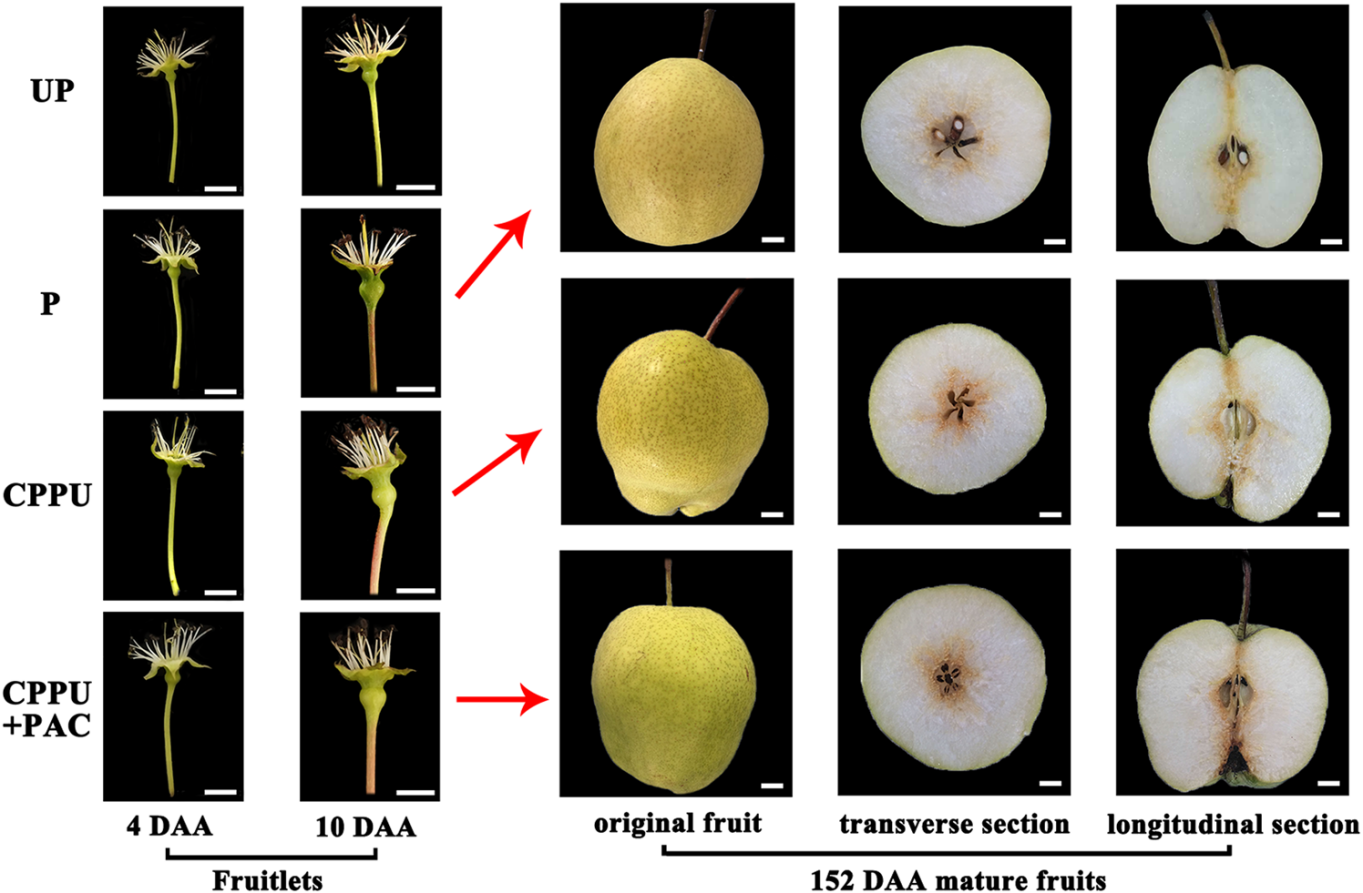
Morphology of ‘Dangshansu’ pear fruits. UP, unpollinated; P, pollinated; CPPU, treatment with *N*-(2-chloro-4-pyridyl)-*N*′-phenylurea alone; CPPU+PAC, treatment with *N*-(2-chloro-4-pyridyl)-*N*′-phenylurea supplemented with paclobutrazol. Fruits were collected at 4, 10, and 152 days after anthesis (DAA). Bar = 1cm

The fruits in each treatment group exhibited morphological differences at 4 DAA. Pollinated fruits and fruits treated with either CPPU alone or PAC-supplemented CPPU appeared to be larger than unpollinated fruits (Fig. 1). Fruits from pollinated, CPPU-alone, and PAC-supplemented CPPU treatment groups were also larger than unpollinated fruits at 10 DAA (Fig. 1). Both CPPU-alone and PAC-supplemented CPPU treatments induced fleshy calyxes at 10 DAA, which led to the formation of fruits that were more deeply lobed (Fig. 1). No mature fruits were produced in the unpollinated group, whereas mature fruits were obtained at 152 DAA from pollinated, CPPU-alone, and PAC-supplemented CPPU treatments. The latter two treatments generated seedless fruits (Supplementary Fig. S1). No significant difference was observed in the weights of pollinated, CPPU-treated, and PAC-supplemented CPPU-treated fruits (Fig. 1; Supplementary Table S1), and parthenocarpic fruits induced by CPPU-alone and PAC-supplemented CPPU treatments were less firm than control fruits (Supplementary Table S1). Moreover, the core transverse diameter of mature seedless fruits was significantly smaller than that of normal fruits (Supplementary Table S1).

**Fig. 2:**
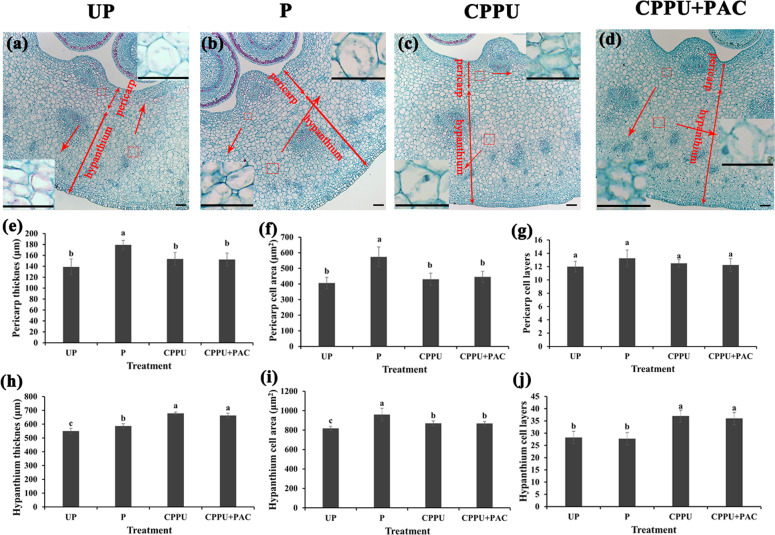
Histological observations of ‘Dangshansu’ pear pericarps and hypanthium at 4 days after anthesis. **a**–**d** Microscopic transverse sections of pericarps and hypanthium of pear fruits after treatments: unpollinated (**a**), pollinated (**b**), treatment with *N*-(2-chloro-4-pyridyl)-*N*′-phenylurea alone (**c**), and treatment with *N*-(2-chloro-4-pyridyl)-*N*′-phenylurea supplemented with paclobutrazol (**d**). Effects of the four treatments on pericarp and hypanthium thickness (**e**, **h**), cell area (**f**, **i**), and number of cell layers (**g**, **j**). Significant differences among treatments as determined by one-way ANOVA (*P*<0.05) are indicated using different lowercase letters. Bar=50μm. Error bars represent the standard deviation of the mean (SD; *n*=9)

To further observe the effects of CPPU treatment on cell development, paraffin slices were prepared from fruits collected at 4 DAA. We found that the pericarp and hypanthium of pollinated fruits were significantly thicker than those of fruits in the unpollinated group, while the CPPU-alone and PAC-supplemented CPPU treatments mainly promoted hypanthium development (Fig. 2a–e, h). Pollination significantly increased the pericarp and hypanthium cell areas (Fig. 2f, i). After treatment with CPPU alone or CPPU supplemented with PAC, the hypanthium cell areas and cell layers were significantly larger than those in the unpollinated treatment (Fig. 2i, j). No significant differences in pericarp and hypanthium thickness, cell area, or the number of cell layers were detected between the CPPU-alone and PAC-supplemented CPPU treatment groups (Fig. 2e–j). These results suggest that CPPU increases hypanthium thickness by promoting cell division and cell expansion at the early fruit development stage and that this process may be independent of gibberellin.

### Phytohormone levels in CPPU-induced parthenocarpic fruits

**Fig. 3:**
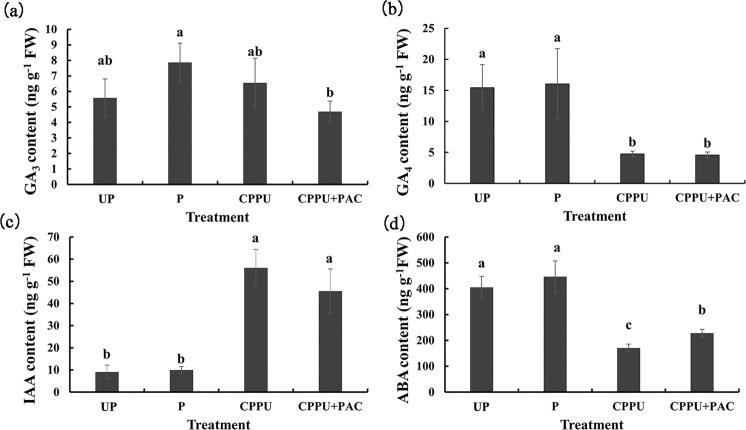
Phytohormone levels in ‘Dangshansu’ pear fruits. Phytohormone levels in ‘Dangshansu’ pear fruits. **a**–**c** Concentrations of the plant phytohormones GA_3_ (**a**), GA_4_ (**b**), IAA (**c**), and ABA (**d**) in fruits collected at 4 days after anthesis. Phytohormone contents are the means of three biological replicates. UP, unpollinated; P, pollinated; CPPU, treatment with *N*-(2-chloro-4-pyridyl)-*N*′-phenylurea alone; CPPU + PAC, treatment with *N*-(2-chloro-4-pyridyl)-*N*′-phenylurea supplemented with paclobutrazol. Significant differences among treatments as determined by one-way ANOVA (*P* < 0.05) are indicated using different lowercase letters. Error bars represent the standard deviation of the mean (SD; *n* = 3)

Because phytohormone application was found to play a key role in promoting parthenocarpy, we measured fruit phytohormone levels. GA_3_ and GA_4_ are the two major active gibberellins in pear. No differences were observed in the GA_3_ contents of fruits from the CPPU-alone and PAC-supplemented CPPU treatment groups compared with that in the unpollinated group at 4 DAA (Fig. 3a). In contrast, the GA_4_ contents of fruits decreased significantly in the CPPU-alone and PAC-supplemented CPPU treatment groups (Fig. 3b). A significant increase in IAA content was detected in the CPPU-alone and PAC-supplemented CPPU treatment groups compared with that in the unpollinated group at 4 DAA, with the IAA content increased significantly, by five-fold, after CPPU treatment (Fig. 3c). In addition, the ABA content of fruits in the CPPU-alone and PAC-supplemented CPPU treatment groups was significantly decreased at 4 DAA (Fig. 3d). These results support our speculation that CPPU-induced parthenocarpy is independent of GA in ‘Dangshansu’ pear. Moreover, IAA and ABA may play important roles in this process.

### Transcriptome analysis of CPPU-induced parthenocarpic fruits

**Fig. 4:**
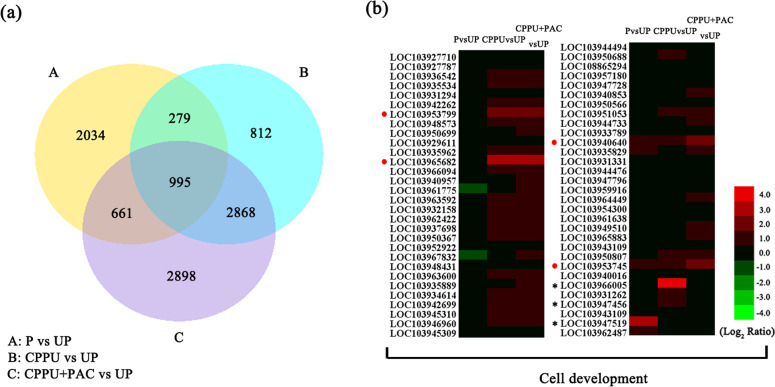
Distribution of differentially expressed genes (DEGs). **a** Distribution of total DEGs in pollinated (P) vs. unpollinated (UP), *N*-(2-chloro-4-pyridyl)-*N*′-phenylurea (CPPU) vs UP, and CPPU supplemented with paclobutrazol (CPPU + PAC) vs. UP treatment groups. **b** Relative expression of cell development-related genes. Circles indicate genes discussed in the text, and asterisks denote genes with FPKM < 1

CPPU took effect rapidly, as cell development and fruit hormone contents among treatment groups exhibited differences at 4 DAA. To investigate the mechanism of CPPU-induced parthenocarpy, we therefore selected fruits collected at 4 DAA for transcriptome analysis. As shown in Supplementary Table S2, 125-bp/150-bp paired-end reads mapped to the pear genome at an average rate of 78%^33^. After mapping sequence reads to the pear genome, we performed a differential expression analysis. This analysis identified 3969, 4954, and 7422 differentially expressed genes (DEGs) present in the pollinated, CPPU-alone, and PAC-supplemented CPPU treatment groups, respectively, compared with the unpollinated group (Fig. 4a). A total of 995 genes were differentially expressed in all three groups (Fig. 4a). To verify the RNAseq results, seven genes that were relevant and/or interesting were selected for qRT-PCR analysis (Supplementary Fig. S2).

### DEGs involved in cell expansion and cell division

On the basis of known functions, 48 and 54 cell development-related DEGs were detected in the CPPU-alone and PAC-supplemented CPPU treatment groups, respectively (Fig. 4b; Supplementary Table S3). After treatment with CPPU alone or PAC-supplemented CPPU, 28 and 33 *cyclin* genes, respectively, were significantly upregulated (Fig. 4b; Supplementary Table S3); among them, 12 were upregulated more than two-fold in both CPPU treatment groups (Fig. 4b; Supplementary Table S3). Genes related to expansins were also upregulated. Three *expansin* genes—*Pbexpansin2* (LOC103940640), *Pbexpansin1* (LOC103953799), and *Pbexpansin-A8* (LOC103953745)—were upregulated nearly four-fold in both the CPPU-alone and PAC-supplemented CPPU treatment groups (Fig. 4b; Supplementary Table S3). Xyloglucan endo-transglucosylases (XTHs) catalyze xyloglucan endohydrolysis and endotransglycosylation, which are processes related to cell expansion. The analysis of the RNA sequencing data revealed that five and four *XTHs* were upregulated more than two-fold after the CPPU-alone and PAC-supplemented CPPU treatments, respectively (Fig. 4b; Supplementary Table S3). *PbXTH32* (LOC103965682) was upregulated eight-fold in both the CPPU-alone and PAC-supplemented CPPU treatment groups (Fig. 4b; Supplementary Table S3). Compared with the CPPU-alone and PAC-supplemented CPPU treatment groups, few cell development-related DEGs were found in the pollinated group, namely, one *cyclin*, three *expansins*, and three *XTHs* were upregulated (Fig. 4b; Supplementary Table S3). These results were consistent with our histological observations.

### DEGs involved in hormone pathways

**Fig. 5:**
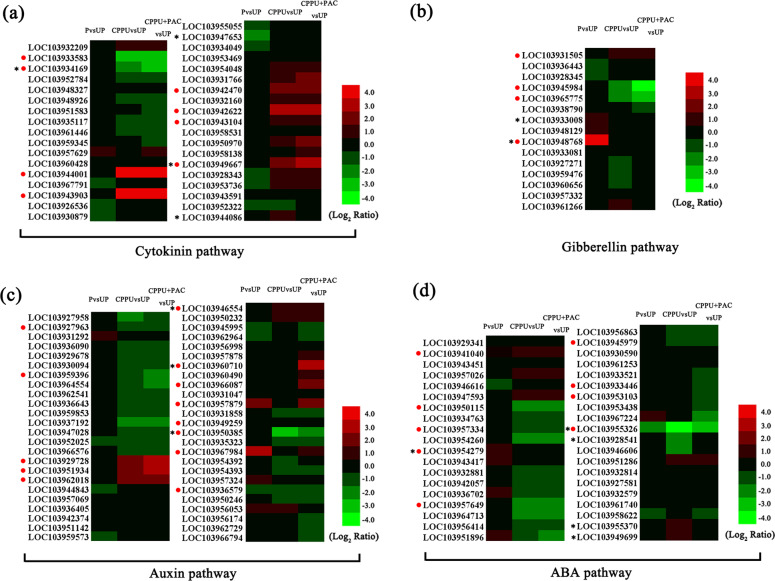
Relative expression of hormone-related genes. (**a**–**d**) Relative expression patterns of gibberellin- (**a**), cytokinin- (**b**), auxin- (**c**), and ABA- (**d**) related genes. UP, unpollinated; P, pollinated; CPPU, treatment with *N*-(2-chloro-4-pyridyl)-*N*′-phenylurea alone; CPPU + PAC, treatment with CPPU supplemented with paclobutrazol. Circles indicate genes discussed in the text, and asterisks denote genes with FPKM < 1

The cytokinin biosynthetic genes *adenylate isopentenyltransferase* (*IPT*) and *cytokinin riboside 5’-monophosphate phosphoribohydrolase* (*LOG*) were significantly downregulated after the CPPU treatment. In particular, *PbIPT5-like* (LOC03934169) was downregulated three-fold, while *PbLOG5* (LOC103933583) was downregulated nearly five-fold (Fig. 5a; Supplementary Table S4). The cytokinin degradation gene *Pbcytokinin dehydrogenase 3-like* (LOC103944001) was upregulated more than 1000-fold in the CPPU-alone and PAC-supplemented CPPU treatment groups (Fig. 5a; Supplementary Table S4). The expression of genes involved in the cytokinin signaling pathway was also altered. *PbAHK4* (LOC103943104), *PbAHK5-like* (LOC103949667), *PbAHP5-like* (LOC103943903), *PbRR9-like* (LOC103942622), and *PbRR10-like* (LOC103942470) were significantly upregulated by treatment with CPPU alone and CPPU supplemented with PAC (Fig. 5a; Supplementary Table S4). *GA20ox* and *GA3ox* are two key genes in the gibberellin biosynthesis pathway. No *GA20ox* or *GA3ox* transcripts were found among the DEGs (Fig. 5b, Supplementary Table S5). These results indicate that CPPU treatment does not induce gibberellin biosynthesis at 4 DAA. *PbGA2ox8* (LOC103948768) was significantly upregulated more than 70-fold in the pollinated group (Fig. 5b; Supplementary Table S5). We also found two *GA2ox* genes (LOC103945984 and LOC103965775) and one *GASA* gene (LOC103931505) that were downregulated and upregulated two-fold, respectively, after both CPPU treatments (Fig. 5b; Supplementary Table S5).

The expression of genes involved in the auxin pathway was altered after the CPPU-alone and PAC-supplemented CPPU treatments. YUCCA is a key enzyme in the auxin biosynthesis pathway. According to the RNA sequencing results, only *PbYUCCA4* (LOC103962018) was upregulated five-fold in both the CPPU-alone and PAC-supplemented CPPU treatment groups (Fig. 5c; Supplementary Table S6). In addition, two transcripts (LOC103960710 and LOC103966087) were significantly upregulated in the PAC-supplemented CPPU treatment groups (Fig. 5c; Supplementary Table S6). After treatment with CPPU and PAC-supplemented CPPU, three auxin-responsive genes—*Pbindole-3-acetic acid-amido synthetase GH3.6* (LOC103946554), *Pbauxin binding protein 19a* (LOC103951934) and *Pbauxin binding protein 20-like* (LOC103929728)—were significantly upregulated more than three-fold, while one auxin-responsive gene—*Pbindole-3-acetic acid-amido synthetase GH3.17-like* (LOC103950385)—was significantly downregulated more than three-fold (Fig. 5c; Supplementary Table S6). Moreover, two auxin-induced genes (LOC103957879 and LOC103967984) were significantly upregulated in the pollinated and PAC-supplemented CPPU treatment groups (Fig. 5c; Supplementary Table S6). In addition, the expression of many ARF and IAA family genes, such as *PbIAA11-like* (LOC103927963), *PbARF3-like* (LOC103936579), *PbARF9* (LOC103949259), and *PbARF19-like* (LOC103959396) (Fig. 5c; Supplementary Table S6), was altered in the pollinated, CPPU-alone, and PAC-supplemented CPPU treatment groups.

According to the RNA sequencing results, three *9-cis-epoxycarotenoid dioxygenases (NCEDs)* (LOC103945979, LOC103955326, and LOC103957334) were downregulated in the CPPU-alone and PAC-supplemented CPPU treatment groups (Fig. 5d; Supplementary Table S7). Among these genes, *PbNCED6* (LOC103955326) was downregulated nearly 20-fold after CPPU treatment (Fig. 5d; Supplementary Table S7). Abscisic acid 8′-hydroxylase is involved in the oxidative degradation of abscisic acid. One transcript (LOC103941040) was upregulated in the pollinated, CPPU-alone, and PAC-supplemented CPPU treatment groups (Fig. 5d; Supplementary Table S7); in addition, one transcript (LOC103954279) was upregulated in the pollinated group, while two transcripts (LOC103950115 and LOC103957649) were downregulated in both the CPPU-alone and PAC-supplemented CPPU treatment groups (Fig. 5d; Supplementary Table S7). Furthermore, eight *protein phosphatase 2Cs* (*PP2Cs*) were downregulated and nine *PP2Cs* were upregulated after the CPPU-alone and PAC-supplemented CPPU treatments (Fig. 5d; Supplementary Table S7). Moreover, some *PP2Cs* were upregulated in the pollinated group and downregulated in the PAC-supplemented CPPU treatment group, such as *PbPP2C23* (LOC103933446) and *PbPP2C49* (LOC103953103) (Fig. 5d; Supplementary Table S7).

### *PbRR9* promotion of *PbYUCCA4* expression and suppression of *PbNCED6* expression

Two-component response regulators (RRs) are important transcription factors in the cytokinin signaling pathway. YUCCA and NCED are key enzymes in the auxin biosynthetic pathway and the ABA biosynthesis pathway, respectively. According to the RNA sequencing results, *PbRR9* (LOC103942622) and *PbNCED6* (LOC103955326) were the most significantly expressed DEGs in the RR and NCED families (Fig. 5b, d; Supplementary Tables S4, S7). The FPKM of *PbRR9* increased from 4 to 50, while that of *PbNCED6* delined from 1 to 0.1 (Fig. S2; Supplementary Table S9). In addition, the only *YUCCA* gene upregulated in both CPPU treatments was *YUCCAA4* (LOC103962018) (Fig. 5c; Supplementary Table S6). *PbRR9*, *PbYUCCA4*, and *PbNCED6* were therefore selected to further evaluate the mechanism of CPPU-induced parthenocarpy.

**Fig. 6:**
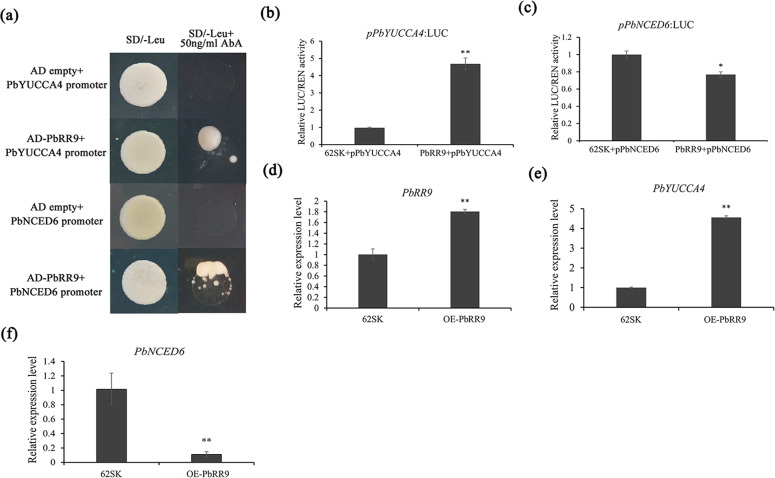
Binding and stimulation of the *PbYUCCA4* and *PbNCED6* promoters by *PbRR9*. **a** Yeast one-hybrid assay showing the interaction of *PbRR9* with the *PbYUCCA4* and *PbNCED6* promoters. **b**, **c** Validation of the activation effect of *PbRR9* on the *PbYUCCA4* (**b**) and *PbNCED6* promoters (**c**) based on a dual luciferase assay. Relative promoter activity is represented by the ratio of the structural gene luciferase (LUC) to 35 S *Renilla* (REN). An empty vector was used as the reference. **d**–**f** The relative expression levels of *PbRR9* (**d**), *PbYUCCA4* (**e**), and *PbNCED6* (**f**) according to a transient assay. Significant differences among treatments were determined by Student’s *t*-test: *P < 0.05; **P < 0.01. Error bars represent the standard deviation of the mean (SD; *n* = 3)

According to the qRT-PCR results, *PbRR9* and *PbYUCCA4* were significantly upregulated and *PbNCED6* was significantly downregulated in the CPPU-alone and PAC-supplemented CPPU treatment groups at 4 DAA (Supplementary Fig. S2). The results of a yeast one-hybrid assay indicated that *PbRR9* can combine with the *PbYUCCA4* and *PbNCED6* promoters (Fig. 6a). Moreover, dual luciferase assays revealed that *PbRR9* can promote the activity of the *PbYUCCA4* promoter and repress the activity of the *PbNCED6* promoter (Fig. 6b, c). To further verify the function of *PbRR9*, we transiently overexpressed *PbRR9* in pear fruits by injection. Successful infection of the ‘Dangshansu’ fruits was confirmed by monitoring GUS signals (Supplementary Fig. S3). qRT-PCR revealed that *PbRR9* overexpression significantly upregulated *PbYUCCA4* and downregulated *PbNCED6* (Fig. 6d–f). These results demonstrate that *PbRR9* is upstream of *PbYUCCA4* and *PbNCED6* and can upregulate *PbYUCCA4* and downregulate *PbNCED6*.

## Discussion

Cytokinin can induce parthenocarpy in many horticultural plants, including watermelon^29^, tomato^30^, grape^31^, and cucumber^32^. Although previous research has shown that CPPU can induce parthenocarpy in pear^23^, the mechanism of induction has been unclear. Parthenocarpy is closely related to the coordinated action of different hormones, among which gibberellin is recognized as a key hormone^5,24,34^. In tomato, CPPU-induced parthenocarpy is partly dependent on enhanced gibberellin and auxin biosynthesis and the application of PAC can significantly decrease gibberellin content^30^. Our previous studies have indicated that GA_4+7_, 2,4-dichlorophenoxyacetic acid (2,4-D), and melatonin (MT) can all induce parthenocarpy, which in pear is dependent on gibberellin^12,24,35^. To investigate whether gibberellin also plays a key role in CPPU-induced parthenocarpy in pear, we sprayed CPPU supplemented with PAC (a gibberellin synthesis inhibitor) on bagged ‘Dangshansu’ pear flowers. We observed no difference in the fruit set rate between the CPPU-alone and PAC-supplemented CPPU treatment groups. This result indicates that blocking the biosynthesis of gibberellin does not inhibit CPPU-induced parthenocarpy in ‘Dangshansu’ pear. We thus speculate that CPPU induces a gibberellin-independent parthenocarpy that is different from that triggered by GA_4+7_, 2,4-D or MT.

After pollination and fertilization, a series of cell division, expansion, and differentiation activities occurs in plants that causes fruit set and development^36,37^. In our study, we found that CPPU can promote cell division and expansion that significantly increases hypanthium thickness and observed that PAC application had no influence on the effect of CPPU on fruit cell development. No differences in hypanthium thickness, cell area, or the number of cell layers were detected between the CPPU-alone and PAC-supplemented CPPU treatment groups. These results suggest that gibberellin does not have an important role in CPPU-induced cell development. The expression of *CDK* and *CYC*, two major gene families involved in cell division, is regulated by hormones^38,39^. EXP and XTH regulate cell size and shape by changing cell wall plasticity^40,41^, while cytokinin affects the expression of cell cycle-related genes and cellular processes to initiate parthenocarpic fruit development^42,43^. According to our RNA sequencing results, several *CYCs*, *CDKs*, and *EXPs* from different subfamilies were upregulated after CPPU-alone and PAC-supplemented CPPU treatment. These results are consistent with histological observations of cell development, which indicates that CPPU promotes cell division and expansion and that the effect of CPPU is not dependent on gibberellin.

Previous research has suggested that GA_3_ and GA_4_ are the major bioactive gibberellins in pear^12,24^. In our study, a phytohormone content assay revealed that neither GA_3_ nor GA_4_ content increased after CPPU treatment, and a decrease even was observed in some cases. Moreover, the RNA sequencing analysis uncovered no *GA20ox* or *GA3ox* genes that were differentially expressed. GA20ox and GA3ox are key enzymes in the gibberellin biosynthesis pathway, and a change in their expression patterns can influence the level of bioactive gibberellins^25^. Thus, CPPU did not promote the synthesis of GA in CPPU-induced parthenocarpy. The above results support our hypothesis that CPPU-induced parthenocarpy in ‘Dangshansu’ pear is gibberellin independent. In addition, we identified two *GA2ox* genes and one *GASA* gene that were downregulated in both CPPU treatment groups. These results may be due to different developmental processes induced by CPPU or to the different tissue localization of different genes^44^.

In addition to our analysis of gibberellins, we found that IAA content was significantly upregulated and ABA content was significantly downregulated after CPPU treatment. Our previous study indicated that a high IAA level and a low ABA level play certain roles in pear parthenocarpy^24^. According to our RNA sequencing results, several genes related to auxin biosynthesis and signaling pathways were differentially expressed after CPPU treatment. *YUCCA* is a key gene in the auxin biosynthesis pathway, and the upregulation of *PbYUCCA4* is consistent with the results of our phytohormone determination. Many genes participating in the auxin signaling pathway, such as *SlARF7*, *SmARF8*, and *SlIAA9*, are related to parthenocarpy^16–18,45^. According to the results of RNA sequencing, *PbARF7* (LOC103929678) was significantly downregulated and *PbIAA9* (LOC103936405) was significantly upregulated in both CPPU treatments. Auxin may thus play an essential role in CPPU-induced parthenocarpy in ‘Dangshansu’ pear. ABA is an important hormone that regulates plant growth and development. The ovaries of parthenocarpic mandarin oranges have low ABA content^21^, which suggests that ABA plays a negative role in fruit set^46^. In our study, three *NCEDs* were significantly downregulated, and several *PP2Cs* were differentially expressed. NCED is a key enzyme in the ABA synthesis pathway^47,48^, and the downregulation of *NCEDs* is consistent with the low ABA levels after the CPPU treatment. *PP2Cs* are regulators of the ABA signaling pathway^49,50^, and the differential expression of *PP2Cs* after CPPU treatment indicates that the ABA pathway is activated by CPPU. ABA may thus also be involved in the process of CPPU-induced parthenocarpy.

To more fully elucidate the mechanism of CPPU-induced parthenocarpy in ‘Dangshansu’ pear, we further analyzed *PbRR9*, *PbYUCCA4*, and *PbNCED6*. Similar to bacterial two-component phosphorelay signal transduction, the cytokinin signaling transduction model has three components: a hybrid HK receptor that contains both histidine kinase and receiver domains, *authentic histidine phosphotransferases (AHPs)* and *separate response regulators (RRs)*^51–53^. RRs play an important role in the cytokinin signaling pathway and regulate the expression of some downstream cytokinin-response genes^53,54^. The results of a yeast one-hybrid assay revealed that *PbRR9* can combine with the *PbYUCCA4* and *PbNCED6* promoters. In addition, dual luciferase assays indicated that *PbRR9* promotes the activity of the *PbYUCCA4* promoter and represses that of the *PbNCED6* promoter. Moreover, we found that *PbYUCCA4* was significantly upregulated and *PbNCED6* was significantly downregulated after transient overexpression of *PbRR9* in fruits. *PbRR9* is an A-type response regulator gene. A-type response regulators are generally regarded as negative regulators of cytokinin signaling^53^; however, a few A-type response regulators have been found to function as positive regulators^55–57^. Furthermore, the expression of *YUCCA* and *NCED* can affect the endogenous IAA and ABA contents^21,58,59^. The data presented here therefore indicate that *PbRR9* is upstream of *PbYUCCA4* and *PbNCED6* and that *PbRR9* can upregulate *PbYUCCA4* and downregulate *PbNCED6* to increase IAA content and decrease ABA content.

Finally, we observed the undeveloped seeds in mature CPPU-induced parthenocarpic fruits. The presence of these seeds suggests that a signal required for the promotion of seed development might be provided by CPPU.

**Fig. 7:**
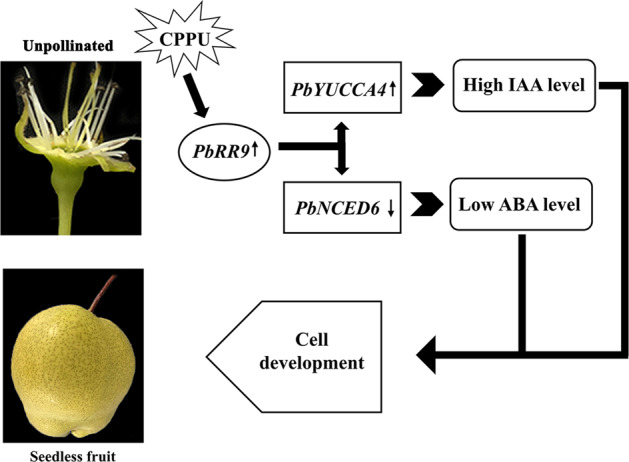
Model depicting the mechanism of N-(2-chloro-4-pyridyl)-N′-phenylurea-induced parthenocarpy in ‘Dangshansu’ pear

In summary, our findings increase our understanding of the novel mechanism of CPPU-induced parthenocarpy (Fig. 7). CPPU may initially upregulate *PbRR9* and then increase IAA content and decrease ABA content by upregulating *PbYUCCA4* and downregulating *PbNCED6*. Cell division and expansion are subsequently activated and eventually induce parthenocarpy in ‘Dangshansu’ pear (Fig. 7). Because fertilization may not have been complete in the pollinated group at 4 DAA, we were unable to compare the differences between normal fruiting and parthenocarpy. In addition, ‘botanical’ and ‘accessory’ fruit structures are different; consequently, the tissues used in this study are different from those in a related study of tomato, which may have led to different conclusions about the mechanisms of CPPU-induced parthenocarpy. The conclusions of our study are therefore still preliminary.

## Materials and methods

### Plant materials and experimental treatments

The experimental plants used in this study were 13-year-old ‘Dangshansu’ pear (*Pyrus bretschneideri* Rehd.) trees in an orchard located in Qianxian, Shaanxi Province, China (34.25° N, 108.12° E; 514 m above sea level). To prevent pollination, we placed bags over the flowers 2 days before anthesis. Four treatments were applied: no pollination (water); pollination; 20 mg l^−1^ CPPU (in water); and 20 mg l^−1^ CPPU supplemented with 5 μmol l^−1^ PAC (in water). Flowers in each treatment group were sprayed at anthesis. Fruits were collected at 4, 10, and 152 days after anthesis (DAA).

### Determination of fruit set rate

When all the flowers in the unpollinated group had fallen off (approximately 20 DAA), we removed the bags and calculated the fruit set rate. A total of 120 blooms on three branches were selected to determine the fruit set rate, which was calculated as follows: fruit set rate (%) = (number of fruits remaining/the total number of fruits) × 100%

### Determination of fruit quality

Ten mature fruits of each group were used to measure fruit quality. The total soluble solids content was determined using a portable system (PAL-1 pocket refractometer, Atago, Japan). Fruit firmness was determined on three opposite sides of the surface of each fruit (equatorial region) using a GY-4 texture analyzer (TOP Instrument, China). A Vernier caliper was used to measure the core transverse diameter. The formula used to calculate the fruit shape index was as follows: fruit shape index = fruit suture diameter/polar diameter.

### Paraffin sectioning

Fruits collected at 4 DAA from unpollinated, pollinated, CPPU, and PAC-supplemented CPPU treatment groups were used as samples (Fig. S4). These samples were immediately fixed in formaldehyde–acetic acid–alcohol fixative^60^ and stored at 4 °C. The fruits were dehydrated in an ethanol/xylene series and embedded in paraffin, sectioned into 8-µm-thick slices, dried, and stained with safranin and fast green. A microscopic imaging system (BX51 + PD72 + IX71, Olympus, Japan) was used to observe the anatomical images. Cell area and tissue thickness were measured using ImageJ software, with three sections from three fruits used for each measurement.

### Phytohormone analysis

Fruits collected at 4 DAA were used as the samples for phytohormone analysis (Supplementary Fig. S4). The fruits (0.5 g) were ground, and 4 ml of extraction buffer (citric acid, 2,6-di-tert-butyl-4-methylphenol, and methyl alcohol) was added to the fruit samples. After shaking overnight at 4 °C, the suspension was centrifuged at 10,000 *g* for 15 min, and the supernatant was withdrawn. Next, 3 ml of extraction buffer was added to the precipitate, which was shaken at 4 °C for 1 h and centrifuged at 10,000 *g* for 15 min. The supernatant was then dried under nitrogen gas, dissolved in 300 µl methanol (0.1% methane acid), and passed through a 0.22-µm filter membrane. GA_3_, GA_4_, IAA, and ABA contents were determined by HPLC–MS/MS (AB SCIEX TripleTOF 5600+, US-MA) using an Acquity UPLC HSS T3 column (1.8 μm; 2.1 × 100 mm; Waters, USA). The injection volume was 2 µl. The MS conditions were as follows: a spray voltage of 4500 V and air curtain, nebulizer, and auxiliary gas pressures of 15, 65, and 70 psi, respectively. The atomizing temperature was 400 °C. Three independent biological replicates were performed.

### Transcriptome analysis

Fruits collected at 4 DAA were used for RNA sequencing (Supplementary Fig. S4). Total RNA was extracted using an RNA Prep Pure Plant kit (Tiangen, Beijing, China). RNA degradation and contamination were monitored on 1% agarose gels, and RNA purity was checked on a NanoPhotometer spectrophotometer (Implen, CA, USA). RNA was quantified using a Qubit RNA Assay kit on a Qubit 2.0 fluorometer (Life Technologies, CA, USA), and RNA integrity was assessed using an RNA Nano 6000 Assay kit supplied with the Bioanalyzer 2100 system (Agilent Technologies, CA, USA). A total of 1 μg RNA per sample was subjected to RNA sequencing on an Illumina HiSeq instrument. Sequencing libraries were generated using a NEBNext Ultra RNA Library Prep kit for Illumina (NEB, USA), with index codes added to attribute sequences to each sample. The clustering of index-coded samples was performed on a cBot cluster generation system using a TruSeq PE cluster kit v3-cBot-HS (Illumina) according to the manufacturer’s instructions. After cluster generation, the library preparations were sequenced on an Illumina HiSeq platform, and 125-bp/150-bp paired-end reads were generated. After constructing a reference genome index in Bowtie v2.2.3, paired-end clean reads were aligned to the reference genome using TopHat v2.0.12. The number of reads mapped to each gene was counted in HTSeq v0.6.1. The RNA-sequencing data from unpollinated fruits were used as controls. A false discovery rate <0.001 and a |log2 ratio| >1 were used as thresholds of the significance of DEGs. Genes were annotated using the ‘Dangshansuli’ database (http://www.ncbi.nlm.nih.gov/genome/?term=pyrus) as a reference. Three independent biological replications were sequenced and analyzed.

### Quantitative real-time PCR (qRT-PCR) validation of gene expression levels

qRT-PCR analysis was performed on a StepOnePlus Real-Time PCR instrument (ABI, US-MA) using TB Green Premix Ex *Taq* II (Tli RNaseH Plus, Takara, Dalian, China). Total RNA was extracted using an RNA Prep Pure Plant kit (Tiangen), and 1 μg of total RNA was then reverse-transcribed to cDNA using a PrimeScript RT reagent kit with gDNA Eraser (Takara). The primers used for qRT-PCR are listed in Supplementary Table S8. *PbActin7* (LOC103926846) was used as a reference gene. Three biological replicates were performed. The relative expression levels of the tested genes were calculated using the cycle threshold (C_t_) 2^−ΔΔC^_t_ method^61^.

### Transient assay

For the overexpression of *PbRR9*, the full-length *PbRR9* (LOC103942622) coding sequence (CDS) was PCR-amplified from ‘Dangshansu’ cDNA sources and then inserted into a pGreenII 0029 62-SK vector using a ClonExpress One Step Cloning kit (Vazyme, Nanjing, China). The constructed vector was expressed under the control of the CaMV 35 S promoter. The complete GUS CDS of the pBI121-GUS plasmid was cloned into the multiple cloning sites (MCS) of the pGreenII 0029 62-SK binary vector to form the pGreenII 0029 62-SK-GUS plasmid. The primers used for CDS amplification are listed in Supplementary Table S8. The resulting plasmid was transferred into *Agrobacterium tumefaciens* strain GV3101 by the heat-shock method, and the *Agrobacterium* cells were then grown at 28 °C in LB solid medium containing appropriate antibiotics (kanamycin and rifampicin). After incubation for 48 h, the *Agrobacterium* cells were resuspended in infiltration buffer (10 mM MgCl_2_, 10 mM MES, and 200 μM acetosyringone) and shaken for 3–4 h at room temperature to achieve a final OD_600_ of 0.8 prior to injection of pear fruits. Twenty-day-old fruits were used for the injection, which was implemented as described in Spolaore et al.^62^ using the injection volume detailed in Zhai et al.^63^. The negative controls were infiltrated with *Agrobacterium* containing a pGreenII 0029 62-SK empty vector. Treated fruits were harvested 5 days after injection. Three independent biological replicates were performed. The GUS staining method was as described in Fillatti et al.^64^.

### Yeast one-hybrid (Y1H) assay

Y1H assays were performed according to the manufacturer’s instructions using a Matchmaker Gold Yeast One-Hybrid System kit (Clontech, Mountain View, CA, USA). To construct pAbAi-baits, we ligated approximately 500-bp fragments of the *PbYUCCA4* (LOC103962018) and *PbNCED6* (LOC103955326) promoters into pAbAi. The complete CDS of *PbRR9* was separately inserted into a pGADT7 vector to construct prey-AD vectors. The pAbAi-bait vectors were linearized and transformed into Y1HGold separately. Colonies were selected on a plate with selective synthetic dextrose medium (SD) lacking uracil. Confirmation of the correct integration of the plasmids into the genome of Y1HGold was performed by colony PCR analysis (Matchmaker Insert Check PCR Mix 1; Clontech). After determining the minimal inhibitory concentration of aureobasidin A (AbA) for the bait reporter yeast strains, the AD-prey vectors were transformed into the bait yeast strains and selected on an SD/-Leu/AbA plate. All transformations and screenings were performed three times.

### Dual luciferase assay

*PbYUCCA4* and *PbNCED6* promoters were amplified from ‘Dangshansu’ genomic DNA using PrimeSTAR Max Premix (Takara) and then cloned into dual-LUC plasmid pGreenII 0800-LUC. The full-length CDS of *PbRR9* was cloned into the MCS (BamHI–HindIII) region of a pGreenII 0029 62-SK binary vector^65^.

Each recombinant plasmid and the pSoup helper plasmid were transferred individually into *Agrobacterium* strain GV3101. *Agrobacterium* cells containing PbRR9-62SK were separately mixed with *PbYUCCA4* promoter-LUC or *PbNCED6* promoter-LUC at a 1:1 ratio before infiltration into 4-week-old *Nicotiana benthamiana* leaves. After injection, the plants were placed in darkness at room temperature for 3 days. The treated leaves were then collected in 1× phosphate-buffered solution for the dual luciferase assay. The ratio of firefly luciferase to *Renilla* luciferase enzyme activities was analyzed using a dual-luciferase reporter assay system (Promega, Madison, WI, USA) on a Tecan Infinite M200pro full-wavelength multifunctional enzyme labeling instrument (TECAN, Hombrechtikon, Switzerland). Three independent biological replicates were performed.

## Supplementary information

supplementary Table 9

Certificate of Editing

Figure S4

Figure S1

Figure S2

Figure S3

Supplementary Table4

Supplementary Table 2

Supplementary Table 1

Supplementary Table 3

Supplementary Table 5

Supplementary Table 6

Supplementary Table 7

Supplementary Table 8

## References

[1] Gillaspy G, Ben-David H, Gruissem W (1993). Fruit-a development perspective. Plant Cell..

[2] Goetz M (2007). Expression of aberrant forms of AUXIN RESPONSE FACTOR8 stimulates parthenocarpy in Arabidopsis and tomato. Plant Physiol..

[3] Gustafson FG (1942). Parthenocarpy: natural and artificial. Bot. Rev..

[4] Ruan YL, Patrick JW, Bouzayen M, Osorio S, Fernie AR (2012). Molecular regulation of seed and fruit set. Trends Plant Sci..

[5] Serrani JC (2010). Inhibition of auxin transport from the ovaries or from the apical shoot induces parthenocarpic fruit-set in tomato mediated by gibberellins. Plant Physiol..

[6] Koshioka M (1994). Analysis of gibberellins in growing fruits of Lycopersicon esculentum after pollination or treatment with 4-chlorophenoxyacetic acid. J. Hort. Sci..

[7] Gustafson FG (1939). Auxin distribution in fruits and its significance in fruit development. Am. J. Bot..

[8] Joldersma D, Liu Z (2018). The making of virgin fruit: the molecular and genetic basis of parthenocarpy. J. Exp. Bot..

[9] Nitsch JP (1950). Growth and morphogenesis of the strawberry as related to auxin. Am. J. Bot..

[10] Serrani JC, Ruiz-Rivero O, Fos M, Garcia-Martinez JL (2008). Auxin-induced fruit-set in tomato is mediated in part by gibberellins. Plant J..

[11] Zhang C, Lee U, Tanabe K (2008). Hormonal regulation of fruit set, parthenogenesis induction and fruit expansion in Japanese pear. Plant Growth Regul..

[12] Cong L (2018). 2,4-D-induced parthenocarpy in pear is mediated by enhancement of GA_4_. Biosynth. Physiol. Plant..

[13] Acciarri, N. et al. Genetically modified parthenocarpic eggplants: improved fruit productivity under both greenhouse and open field cultivation. *BMC Biotechnol*. **2**, 4 (2002). 10.1186/1472-6750-2-4 PMC101493 11934354

[14] Rotino GL, Perri E, Zottini M, Sommer H, Spena A (1997). Genetic engineering of parthenocarpic plants. Nat. Biotechnol..

[15] Ren Z (2011). The auxin receptor homologue in Solanum lycopersicum stimulates tomato fruit set and leaf morphogenesis. J. Exp. Bot..

[16] De Jong M, Wolters-Arts M, Feron R, Mariani C, Vriezen WH (2009). The Solanum lycopersicum auxin response factor 7 (SlARF7) regulates auxin signaling during tomato fruit set and development. Plant J..

[17] Wang H (2005). The tomato Aux/IAA transcription factor *IAA9* is involved in fruit development and leaf morphogenesis. Plant Cell..

[18] Mazzucato A (2015). A TILLING allele of the tomato Aux/IAA9 gene offers new insights into fruit set mechanisms and perspectives for breeding seedless tomatoes. Mol. Breed..

[19] Watanabe M, Segawa H, Murakami M, Sagawa S, Komori S (2008). Effects of plant growth regulators on fruit set and fruit shape of parthenocarpic apple fruits. J. Jpn. Soc. Hortic. Sci..

[20] Prosser MV, Jackson GAD (1959). Induction of parthenocarpy in Rosa arvensis huds with gibberellic acid. Nature.

[21] Mesejo C, Reig C, Martínez-Fuentes A, Agustí M (2010). Parthenocarpic fruit production in loquat (Eriobotrya japonica Lindl.) by using gibberellic acid. Sci. Hortic..

[22] Jung CJ (2014). Transcriptional changes of gibberellin oxidase genes in grapevines with or without gibberellin application during inflorescence development. J. Plant Res..

[23] Niu QF (2015). Effects of exogenous application of GA_4+7_ and N-2-chloro-4-pyridyl-N’-phenylurea on induced parthenocarpy and fruit quality in Pyrus pyrifolia ‘Cuiguan’. Plant Growth Regul..

[24] Liu LL (2018). Histological, hormonal and transcriptomic reveal the changes upon gibberellin-induced parthenocarpy in pear fruit. Hortic. Res..

[25] Giacomelli L (2013). Gibberellin metabolismin Vitis vinifera L. during bloom and fruit-set: functional characterizationand evolution of grapevine gibberellin oxidases. J. Exp. Bot..

[26] García-Hurtado N (2012). The characterization of transgenic tomato overexpressing gibberellin 20-oxidase reveals induction of parthenocarpic fruit growth, higher yield, and alteration of the gibberellin biosynthetic pathway. J. Exp. Bot..

[27] Martinez-Bello L, Moritz T, Lopez-Diaz I (2015). Silencing C19-GA 2-oxidases induces parthenocarpic development and inhibits lateral branching in tomato plants. J. Exp. Bot..

[28] Martí C (2007). Silencing of DELLA induces facultative parthenocarpy in tomato fruits. Plant J..

[29] Hayata Y, Niimi Y, Iwasaki N (1995). Synthetic cytokinin-1-(2=chloro=4=pyridyl)-3-phenylurea (CPPU)-promotes fruit set and induces parthenocarpy in watermelon. J. Am. Soc. Hortic. Sci..

[30] Ding J (2013). Cytokinin induced parthenocarpic fruit development in tomato is partly dependent on enhanced gibberellin and auxin biosynthesis. PLoS ONE.

[31] Lu L (2016). Auxin- and cytokinin-induced berries set in grapevine partly rely on enhanced gibberellin biosynthesis. Tree Genet. Genomes.

[32] Qian C (2017). Effects of exogenous application of CPPU, NAA and GA_4+7_ on parthenocarpy and fruit quality in cucumber (*Cucumis sativus* L.). Food Chem..

[33] Wu J (2013). The genome of the pear (*Pyrus bretschneideri* Rehd.). Genome Res..

[34] Tang N, Deng W, Hu G, Hu N, Li Z (2015). Transcriptome profiling reveals the regulatory mechanism underlying pollination dependent and parthenocarpic fruit set mainly mediated by auxin and gibberellin. PLoS ONE.

[35] Liu JL (2018). Melatonin induces parthenocarpy by regulating genes in gibberellin pathways of ‘Starkrimson’ pear (*Pyrus communis* L.). Front Plant Sci..

[36] Vivian-Smith A, Koltunow AM (1999). Genetic analysis of growth-regulator-induced parthenocarpy in Arabidopsis. Plant Physiol..

[37] Seymour GB, Granell A (2013). Fruit development and ripening. Annu. Rev. Plant Biol..

[38] Inzé D, Veylder LD (2006). Cell cycle regulation in plant development. Annu. Rev. Genet..

[39] Mesejo C (2016). Gibberellin reactivates and maintains ovary-wall cell division causing fruit set in parthenocarpic Citrus species. Plant Sci..

[40] Marowa P, Ding A, Kong Y (2016). Expansins: roles in plant growth and potential applications in crop improvement. Plant Cell Rep..

[41] Atkinson RG, Johnston SL, Yauk Y, Sharma NN, Schröder R (2009). Analysis of *xyloglucan endotransglucosylase/hydrolase* (*XTH*) gene families in kiwifruit and apple. Postharvest Biol. Tec..

[42] Fu FQ, Mao WH, Shi K, Zhou YH, Yu JQ (2010). Spatio-temporal changes in cell division, endoreduplication and expression of cell cycle-related genes in pollinated and plant growth substances-treated ovaries of cucumber. Plant Biol..

[43] Matsuo S, Kikuchi K, Fukuda M, Honda I, Imanishi S (2012). Roles and regulation of cytokinins in tomato fruit development. J. Exp. Bot..

[44] Li C (2019). Comprehensive expression analysis of Arabidopsis GA2-oxidase genes and their functional insights. Plant Sci..

[45] Du L (2016). *SmARF8*, a transcription factor involved in parthenocarpy in eggplant. Mol. Genet. Genomes.

[46] Vriezen WH, Feron R, Maretto F, Keijman J, Mariani C (2008). Changes in tomato ovary transcriptome demonstrate complex hormonal regulation of fruit set. N. Phytol..

[47] Luchi S (2001). Regulation of drought tolerance by gene manipulation of 9-cisepoxycarotenoid dioxygenase, a key enzyme in abscisic acid biosynthesis in Arabidopsis. Plant J..

[48] Lefebvre V (2006). Functional analysis of Arabidopsis NCED6 and NCED9 genes indicates that ABA synthesized in the endosperm is involved in the induction of seed dormancy. Plant J..

[49] Sheen J (1998). Mutational analysis of protein phosphatase 2C involved in abscisic acid signal transduction in higher plants. Proc. Natl. Acad. Sci. USA.

[50] Han, S. et al. Modulation of aba signaling by altering vxgΦl motif of pp2cs in, oryza sativa. *Mol. Plant* **10**, 1190–1205 (2017). 10.1016/j.molp.2017.08.003 28827170

[51] Higuchi M (2004). In planta functions of the Arabidopsis cytokinin receptor family. Proc. Natl. Acad. Sci. USA.

[52] Schaller GE, Shiu SH, Armitage JP (2011). Two-component systems and their co-option for eukaryotic signal transduction. Curr. Biol..

[53] Kieber JJ, Schaller GE (2018). Cytokinin signaling in plant development. Development.

[54] Zubo Y (2017). Cytokinin induces genome-wide binding of the type-B response regulator ARR10 to regulate growth and development in Arabidopsis. Proc. Natl. Acad. Sci. USA.

[55] Osakabe Y (2002). Overexpression of Arabidopsis response regulators, ARR4/ATRR1/IBC7 and ARR8/ATRR3, alters cytokinin responses differentially in the shoot and in callus formation. Biochem. Biophys. Res. Commun..

[56] Leibfried A (2005). WUSCHEL controls meristem function by direct regulation of cytokinin-inducible response regulators. Nature.

[57] Lee BH (2009). Studies of aberrant phyllotaxy1 mutants of maize indicate complex interactions between auxin and cytokinin signaling in the shoot apical meristem. Plant Physiol..

[58] Ji K (2012). Non-climacteric ripening in strawberry fruit is linked to ABA, *FaNCED2* and *FaCYP707A1*. Funct. Plant Biol..

[59] Wang Y, Ding G, Gu T, Ding J, Li Y (2017). Bioinformatic and expression analyses on carotenoid dioxygenase genes in fruit development and abiotic stress responses in Fragaria vesca. Mol. Genet. Genomics.

[60] Henwood A (2010). What is the best procedure to remove formalin pigment from formalin pigment from formaldehyde-acetic acid-alcohol fixed tissues?. J. Histotechnol..

[61] Livak KJ, Schmittgen TD (2001). Analysis of relative gene expression data using real-time quantitative PCR and the 2^−ΔΔCt^ method. Methods.

[62] Spolaore S, Trainotti L, Casadoro G (2001). A simple protocol for transient gene expression in ripe fleshy fruit mediated by Agrobacterium. J. Exp. Bot..

[63] Zhai R (2016). Two MYB transcription factors regulate flavonoid biosynthesis in pear fruit (Pyrus bretschneideri Rehd.). J. Exp. Bot..

[64] Fillatti JJ, Kiser J, Rose R, Comai L (1987). Efficient transfer of a glyphosate tolerance gene into tomato using a binary agrobacterium tumefaciens vector. Nat. Biotechnol..

[65] Hellens RP (2005). Transient expression vectors for functional genomics, quantification of promoter activity and RNA silencing in plants. Plant Methods.

