# Supplementary material for: CPPU may induce gibberellin-independent parthenocarpy associated with PbRR9 in ‘Dangshansu’ pear
Source: Hortic Res. 2020 May 1;7:68. doi: 10.1038/s41438-020-0285-5 (PMC7192895; doi:10.1038/s41438-020-0285-5)
Supplement: Supplementary file 2 — Certificate of Editing [file 41438_2020_285_MOESM2_ESM.pdf]

理文编辑

Certificate of Editing

Edited provisional title

CPPU may induce gibberellin-independent parthenocarpy associated with PbRR9 in ‘Dangshansu’ pear

Client name and institution

Liu Cong, College of Horticulture, Northwest A&F University Taicheng Road No.3, Yangling, Shaanxi Province, China

Date Completed
2020-02-06

Identification code
77211

Certificate issued by
Koji Yamashita
Managing Director and CEO

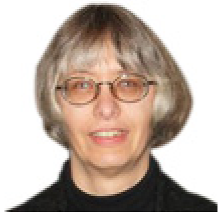

Expert Editor: Barbara Goodson
2007 PhD Botany
University of Texas
Ecology, Genetics, Plant Biology

www.liwenbianji.cn
While this certificate confirms the authors have used Edanz's editing services, we cannot guarantee that additional changes have not been made after our edits.
