# Supplementary figures and images for: CPPU may induce gibberellin-independent parthenocarpy associated with PbRR9 in ‘Dangshansu’ pear
Source: Hortic Res. 2020 May 1;7:68. doi: 10.1038/s41438-020-0285-5 (PMC7192895; doi:10.1038/s41438-020-0285-5)

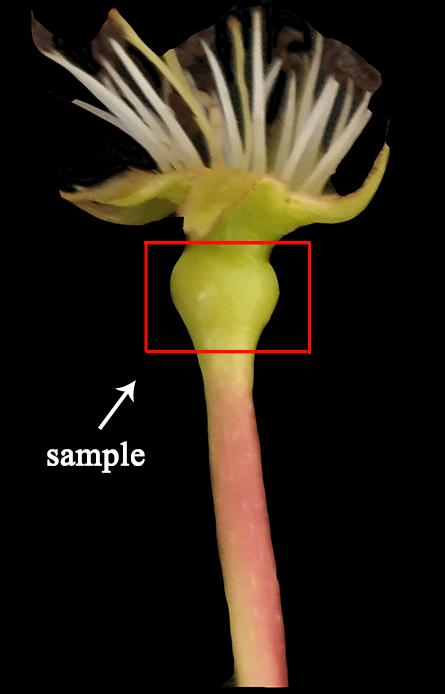

Supplement: Supplementary file 3 — Figure S4 [file 41438_2020_285_MOESM3_ESM.tif]

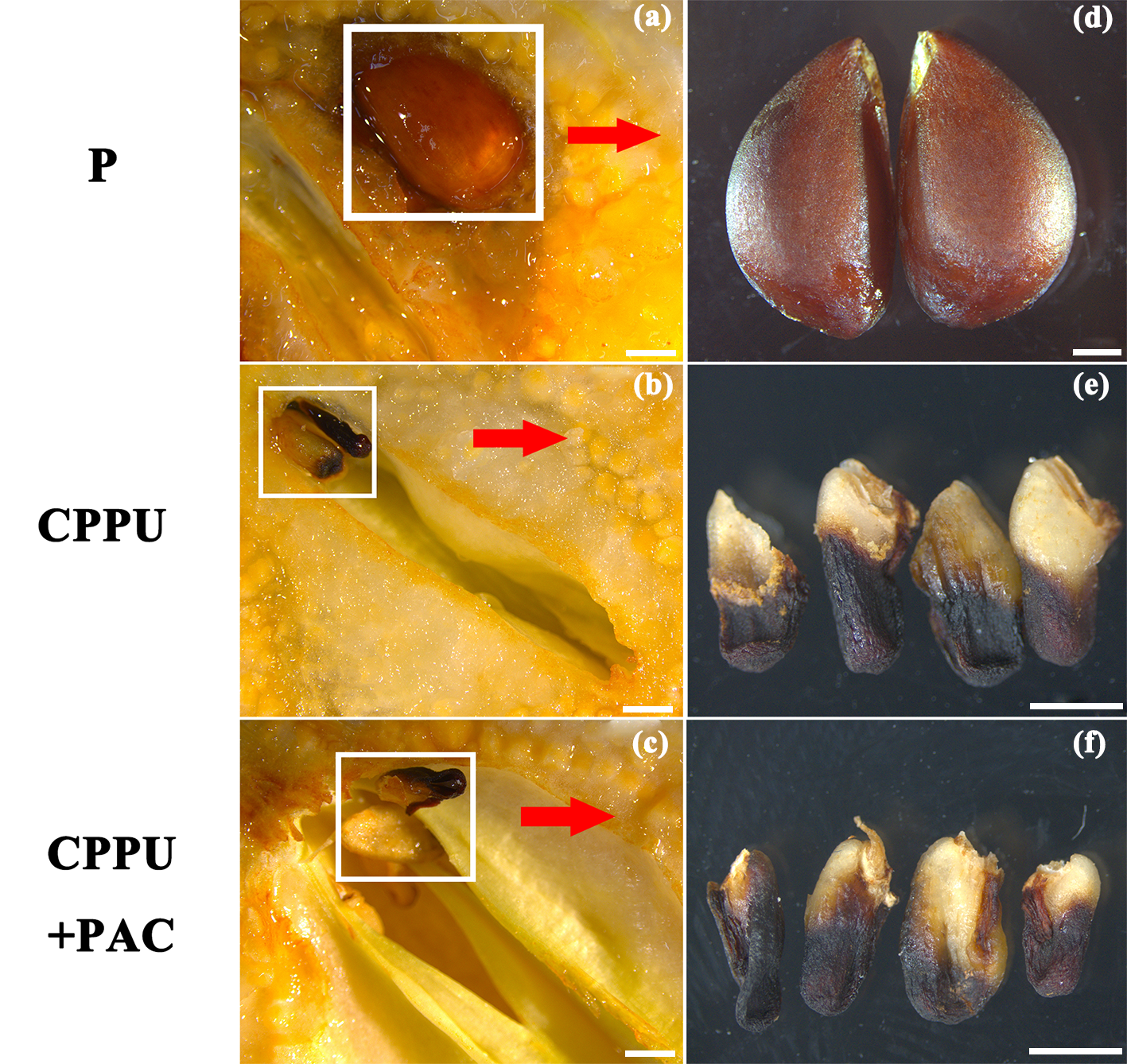

Supplement: Supplementary file 4 — Figure S1 [file 41438_2020_285_MOESM4_ESM.tif]

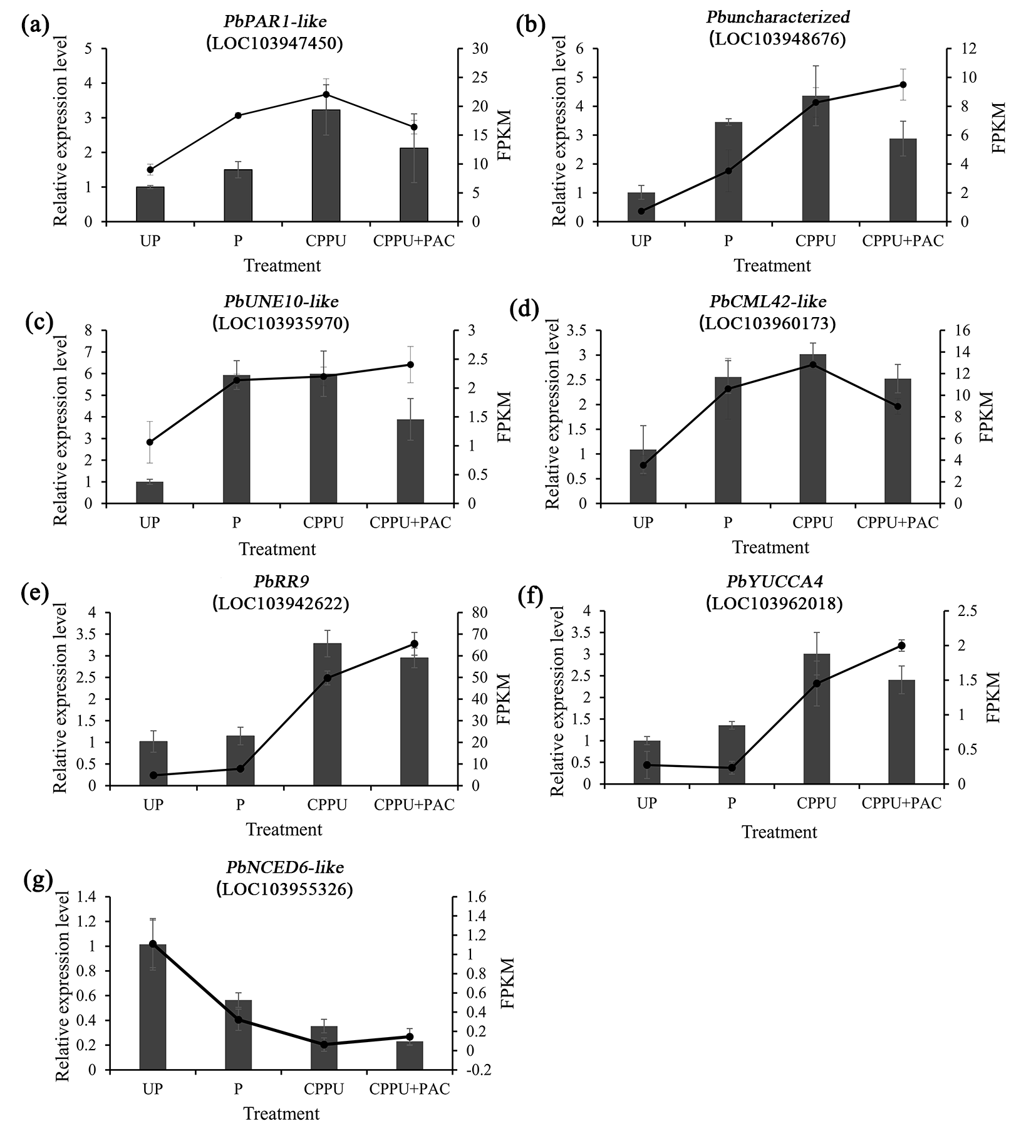

Supplement: Supplementary file 5 — Figure S2 [file 41438_2020_285_MOESM5_ESM.tif]

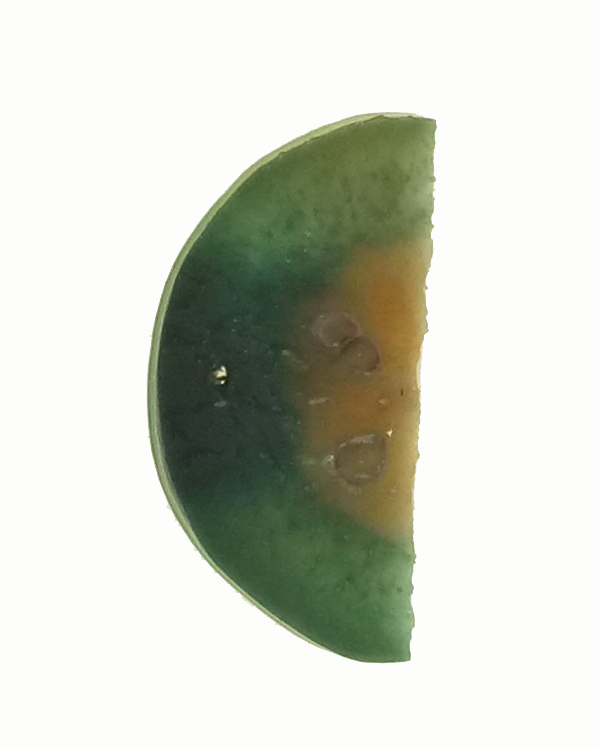

Supplement: Supplementary file 6 — Figure S3 [file 41438_2020_285_MOESM6_ESM.tif]
